# Clinical chemistry laboratory errors at St. Paul’s Hospital Millennium Medical College (SPHMMC), Addis Ababa, Ethiopia

**DOI:** 10.1186/s13104-018-3893-5

**Published:** 2018-11-03

**Authors:** Hirut Tadesse, Kassu Desta, Samuel Kinde, Fatuma Hassen, Addisu Gize

**Affiliations:** 1 Department of Laboratory Science, St. Paul’s Hospital Millennium Medical College, Addis Ababa, Ethiopia; 2 Department of Medical Laboratory Sciences, School of Allied Health Sciences, College of Health Sciences, Addis Ababa University, Addis Ababa, Ethiopia; 3 Department of Microbiology, St. Paul’s Hospital Millennium Medical College, Addis Ababa, Ethiopia

**Keywords:** Clinical chemistry laboratory errors, Pre-analytical, Analytical, Post-analytical errors

## Abstract

**Objective:**

This study was aimed to determine the magnitude of errors in clinical chemistry laboratory tests at different phases of the assay of clinical chemistry laboratory unit.

**Results:**

From the total 1633 clinical chemistry laboratory tests done, overall, 541 (33.1%) errors occurred which accounts that 392 (72.3%), 45 (8.3%), and 104 (19.2%) were pre analytical, analytical and post analytical phases of errors, respectively. Incomplete clinical data of patient was observed on 1185 (72.6%) of CLL tests. Name, gender, and age of patients were missed on 8 (0.5%), 190 (11.6%), and 257 (15.7%) forms of the requests, respectively. The physician’s name existed only on 248 (15.2%) and signature on 1137 (69.6%) of the request forms. An essential patient data were incomplete, which needs emphasis on awareness creation. Such practice improves laboratory data interpretation and thereby prevent misdiagnose and mistreatment of patients.

## Introduction

Analysis of body fluids in clinical chemistry laboratory (CCL) is subjected to a number of interferences that affect the pre analytical and analytical accuracy. The interference arises from exogenous sources like drugs and additives as well as such endogenous sources like lipemia, hemolysis and icteria. The clinical chemistry laboratory unit personnel and clinician should constantly be aware of this factor [1, 2].

Some of the procedures performed within the laboratory include verifying laboratory results, feeding them into the laboratory information system, and communicating them to the clinicians in a number of ways in particular, by producing a report and making any necessary oral communications regarding ‘‘alert’’ or panic results. Study of laboratory errors in an India Hospital mentioned earlier reported post analytical errors as higher as 16% of all laboratory errors [3, 4].

Evaluation of errors in clinical biochemistry laboratory in New Delhi India showed total error rate of 1.4%, which had contribution of 77.1% (pre-analytical), 7.9% (analytical) and 15% (post-analytical) [5, 6]. Another study identified 189 laboratory errors, a relative frequency of 0.47%, which makes distribution of mistakes was pre-analytical 68.2%, analytical 13.3%, and post analytical 18.5% [7–9].

Different studies showed different proportions of rejection specimens; highest in the inpatient services (47.15%) followed by Emergency Department (ED) and outpatient service with 27.40% and 25.39% respectively [10] and 67.4% pre-analytic errors were recorded in Italy [11]. Measuring CCL related errors are mandatory for the total quality of laboratory information to be more effective for patient management; diagnosis and treatment of disease, clinical monitoring and disease prevention. However, studies are non-existent to get the required information in the study area, or in our country at large. So, the main objective of this study was to assess the magnitude of laboratory errors in clinical chemistry laboratory at St. Paul’s Hospital Millennium Medical College, Addis Ababa, Ethiopia.

## Main text

### Methods

Cross sectional study design was conducted from December 2015 to March 2016 at St. Paul’s Hospital Millennium Medical College (SPHMMC), Addis Ababa, Ethiopia. Many patients are referred from different parts of the country and the hospital performs different services or disciplines as needed, available and serve on the average around 800 patients daily. Blood drawing and sample collections are performed by physicians and nurses from the individual wards and specimens are transported manually by ward staff, laboratory runners and also patient’s family to the laboratory.

Firstly, pre-analytical phase’s errors include all processes from the time a laboratory request is made by the physician until the sample is ready for testing. The main processes that should be taken into account in the study of the pre-analytical phase are; test selection, patient preparation, collection, transport, handling and preservation of the samples. Secondly, some of the errors in analytical phase include equipment malfunction, sample mix-ups, and interference (endogenous or exogenous) which could be classified into random errors and systematic errors.

Thirdly, in the post-analytical errors phase of the testing process, results are released to the clinician, and she/he interprets them and makes diagnostic and therapeutic decisions accordingly. In post analytical step, delayed and incorrect results or report those results to whom did not request the laboratory result are the most frequently existed errors.

All clinical chemistry laboratory samples requests were included during the study and requests like blood samples for hematological analysis, stool examination and urinalysis test requests were excluded. Based on the check list, patient socio-demographic information, clinical detail of the patient are collected and observation was made for the presence or absence of hemolysis or sample volume.

Pre-tested check list was used for data collection to increase the quality of data; in addition training was given for data collectors. The data was collected by two trained laboratory technicians during routine and duty hours. The collected data were also checked for completeness by the principal investigator. Data were cleaned and entered into the computer using Excel sheet and exported to SPSS version 20 for analysis. P values less than 0.05 were considered statistically significant.

Ethical approval was obtained from Department of Ethics and Review Committee (DERC), College of Health Science, Addis Ababa University. Based on the approval of the DERC, informed written permission was obtained from St. Paul’s Hospital Millennium Medical College Institutional Review Board (IRB) and submitted to the head of the laboratory department. Informed consent was taken from each participant. Any data generated from the specimens protected the patent privacy, confidentiality and anonymity.

### Results

One thousand six hundred thirty three (1633) clinical chemistry request forms were examined.

From the total figure received in CCL, 828 (50.7%) of them were female, 622 (38%) of the specimen were male patients and the remaining 183 (11.2%) of the requests gender was not specified. Overall, 541 (33.1%) errors were found. The highest well documented parameters were the patient identification number of the requests 1633 (100%) and the information about the cite location of requested clinician’s, 1615 (98.8%). Uncompleted patient’s laboratory request forms may affect interpretation of test results, the most parameter errors which observed in the study were missing of writing physician’s name, 1385 (84.8%) and clinical data of the patient, 1185 (72.6%) (Table 1).

**Table 1:** Total pre-analytical errors observed (N = 1633) on clinical chemistry laboratory request forms in SPHMMC, Addis Ababa, Ethiopia

Items	Well written (n)	% well written	Errors (n)	% errors
Patient ID number	1633	100	–	–
Patient name	1625	99.5	8	0.5
Gender	1376	84.3	257	15.7
Age	1443	88.4	190	11.6
Ward/clinic name	1615	98.9	18	1.1
Physician name	248	15.2	1385	84.8
Clinical data	448	27.4	1185	72.6
Physician signature	1137	69.6	496	30.4
Correct request	1387	84.9	246	15.1
Date of request	1474	90.3	159	9.7

#### Pre-analytical, analytical and post-analytical errors

From the overall, 541 (33.1%) errors, the contribution of the different phases towards the total number of errors were 72.3% (pre-analytical), 8.3% (analytical) and 19.2% (post-analytical). The most common errors were insufficient blood volume from pre-analytical phase, equipment malfunction from analytical phase and communication errors from post-analytical phases (Table 2).

**Table 2:** Pre-analytical, analytical and post-analytical percent of errors in clinical chemistry laboratory, at SPHMMC, Addis Ababa, Ethiopia, N = 541

Pre-analytic phase	N (%)	Analytic phase	N (%)	Post-analytic phase	N (%)
Inadequate sample	220 (40.6)	Equipment malfunction	16 (2.95)	Communication	75 (14)
Hemolyzed sample	124 (22.9)	QC incompatibility	16 (2.95)	Transcription	6 (1.1)
Lipemic sample	25 (4.6)	Reagent expired	7 (1.3)	Data entry	17 (3.1)
Icterus sample	15 (2.8)	Reagent contamination	1 (0.2)	Sample delay	4 (0.7)
Over volume	9 (1.7)	Reagent storing	4 (0.7)	Loss of results	2 (0.4)
Total	393 (73)	Total	44 (8.3)	Total	104 (19)

Logistic analysis revealed communication error with laboratory test requested location cite from OPD, and inpatient had significance association. The occurrence of laboratory results not collected by responsible body increased by three times in OPD from others (Table 3).

**Table 3:** The post-analytical Clinical chemistry error the association between communication errors to the location of laboratory request in SPHMMC, Addis Ababa, Ethiopia, N = 75

Location	Communication		Crude OR	P value	Adjusted OR	P value
	Yes	No	95% CI		95% CI	
OPD						
Yes	44	23	0.32 (0.11–0.95)	0.027	3.10 (1.06–9.08)	0.039
No	31	5	1:00			
Inpatient						
Yes	26	2	6.63 (1.46–30.23)	0.004	0.151 (0.33–0.68)	0.014
No	49	25	1:00			
Emergency						
Yes	4	3	0.45 (0.09–2.16)	0.27	2.22 (0.46–10.63)	0.319
No	71	24	1:00			

*OPD* outpatient department

### Discussion

Across-sectional study conducted in Indian showed, pre-analytical errors affecting the laboratory results. No diagnosis was provided on 61.20% of the laboratory request forms. Type of specimen was not mentioned in 61.60% of the forms and 89.25% of all request forms were illegible [12]. Also, in Nigeria, in 2012 their data showed that the mostly omitted information was the patient’s age, observed in 48.3% of request forms reviewed [13].

Laboratory quality is one of the big issues in our context because the laboratory medicine plays a pivotal role in the provision of health care services. In the present study, errors were detected in 542 sample requests, with a total error rate of 33.1%. Out of which the total error pre-analytical (24%), analytical (2.8%) and post analytical phases (6.4%) which contribute a frequency of 72.3%, 8.3% and 19.2%, respectively. The computable research done in New Delhi; India [14] with a total error rate of 1.4%, pre analytical, analytical, post analytical phases contributed to 1.1%, 0.1% and 0.2% of errors respectively as the contribution of the different phases towards the total number of error gave 77.1% (pre-analytical errors), 7.9% (analytical errors) and 15% (post-analytical errors).

From the total of 1633 clinical chemistry laboratory requests, physician name and signatures were missed in 1385 (84.8%) and 469 (30.3%), respectively. This leads to getting additional information about the patient status, or addressing of laboratory test results the corresponding clinicians show serious drawbacks by the omission of that information. Clinical data not written on the request of 1185 (72.6%), it was higher frequency as compared to the study of India among 1513 request evaluation of which 61.25% of the request of clinical data were missed [15], the variation may be attributed high workload and poor documentation in our situation. We found the highest prevalence of errors 72.5% (393/542) in the pre-analytical phase. Insufficient volume blood sample drawn was the most common error for unsuitable specimen 220 (40.6%) samples. The next most common cause of error was due to incorrect procedures for hemolysis sample collection at 124 (22.8%). The result was lower than the Indian study as its indication of common errors of hemolysis was 53.2% whereas higher lipemia 25 (4.6%) samples in the current study were observed [14]. These discrepancies may be due to lack of proper orientation of patient’s to collect the sample and preparation of sample collectors. In addition the high figure of pre-analytical errors may have partly depended on the fact that most originated from all wards/departments, and not only in the laboratory.

In the current study, 44 errors of events were identified in the analytical phases; which contribute for 8.3% of the total errors. The most frequently detected analytical problems were due to equipment malfunction 16 (35.6%) and non-conformity with QC 16 (35.6%) of the total error frequency. Other sources of analytical errors were reagent expiry 7 (15.6%), calibration drift 1 (2.2%) and contamination of reagents 1 (2.2%). The finding was comparable to the Indian study [14]. From a total 104 (19%) post-analytical errors; communication errors contribute to 75 (72.1%), data entry errors 17 (16.3%), transcription errors 6 (5.8%), delay sample 4 (0.7%). This total post analytical phase errors also comparable again with the errors observed in India 143 (14.9%) [14], and communication error contributed to the majority of post analytical errors 75 (14%).

### Conclusion

In the present study most errors are occurred before samples were analyzed during pre-analytical phase; particularly inadequate sample collection procedure and communication error from OPD department in post analytical phase. This suggests that providing sample collection procedure manual for those who involved in laboratory sample collection and coordination between laboratory and OPD staff workers are the key points for the improvement clinical chemistry laboratory service.

## Limitation of the study

Our study has some limitation like lack of getting similar studies done in Ethiopia, which made difficult for getting more information on the sample size. In the case of some variables; like hemolysis and icterus samples, measurements were made by visual observation which may lead to interpersonal bias, and also we used trained laboratory technician staff as a data collectors which may introduce a social desirability bias.

## Abbreviations

CCL: clinical chemistry laboratory
QC: quality control
SPHMMC: St. Paul’s Hospital Millennium Medical College
SPSS: Statistical Package for Social Science
